# Designing Colloidal Molecules with Microfluidics

**DOI:** 10.1002/advs.201600012

**Published:** 2016-04-25

**Authors:** Bingqing Shen, Joshua Ricouvier, Florent Malloggi, Patrick Tabeling

**Affiliations:** ^1^ MMNESPCIUMR Gulliver10 rue Vauquelin75005ParisFrance; ^2^ UMR 3299 CEA/CNRS NIMBE‐LIONSCEA Saclay91191Gif‐sur‐YvetteFrance

**Keywords:** building blocks, colloidal molecules, high throughput, microfluidics, self‐assembly

## Abstract

The creation of new colloidal materials involves the design of functional building blocks. Here, a microfluidic method for designing building blocks one by one, at high throughput, with a broad range of shapes is introduced. The method exploits a coupling between hydrodynamic interactions and depletion forces that controls the configurational dynamics of droplet clusters traveling in microfluidic channels. Droplet clusters can be solidified in situ with UV. By varying the flow parameters, clusters are prescribed a given size, geometry, chemical and/or magnetic heterogeneities enabling local bonding. Compact structures (chains, triangles, diamonds, tetrahedrons,...) and noncompact structures, such as crosses and T, difficult to obtain with current techniques are produced. Size dispersions are small (2%) and throughputs are high (30 000 h^−1^). The work opens a new pathway, based on microfluidics, for designing colloidal building blocks with a potential to enable the creation of new materials.

## 1 Introduction

In the field of colloidal science, the efforts undertaken over the last ten years have led to design a variety of colloidal structures with a potential to serve as building blocks for new materials.^1^, ^2^, ^3^, ^4^, ^5^, ^6^, ^7^, ^8^ Today, the most promising methods used for creating building blocks, being based on bulk techniques, achieve high throughputs, but, in the meantime, suffer from limitations in terms of control, dispersity, process complexity, yield, and ranges of shapes. Interesting structures, such as colloidal chiral “molecules” or noncompact clusters are difficult to design with these techniques. These limitations are not viewed as critical at the moment, but they complicate or jeopardize the creation of functional colloidal materials. There is a need to forge new pathways toward designing colloidal structures that can enrich the actual library of building blocks, and facilitate or enable the creation of functional colloidal materials, which today represents one of the most important challenges of colloidal science.

In this context, microfluidics might play a valuable role. The short characteristic times resulting from the physics of small systems favor high throughputs. The deterministic nature of the process, linked to low Reynolds numbers, guarantees excellent control, reproducibility, and monodispersity. However, using microfluidic technology to design colloidal building blocks is challenging. How is it possible, with this technology, to drive particles at the microscale to obtain clusters of interesting configurations? In fact, the attempts to improve cluster production with microfluidics^9^, ^10^, ^11^, ^12^ have resulted in a modest success. Recent theoretical work has suggested to form arbitrary assemblies of particles by imposing specific sequences of pressures steps in microfluidic chambers.^13^, ^14^ However, this concept is still awaiting experimental support.

In the present work, we report a new microfluidic strategy for designing monodisperse colloidal “molecules”, one by one, with a broad range of controlled shapes, with a potential to serve as building blocks for colloidal materials. The approach exploits an interesting physical mechanism that has not yet been reported in the literature.

## 2 Cluster Formation

**Figure 1:**
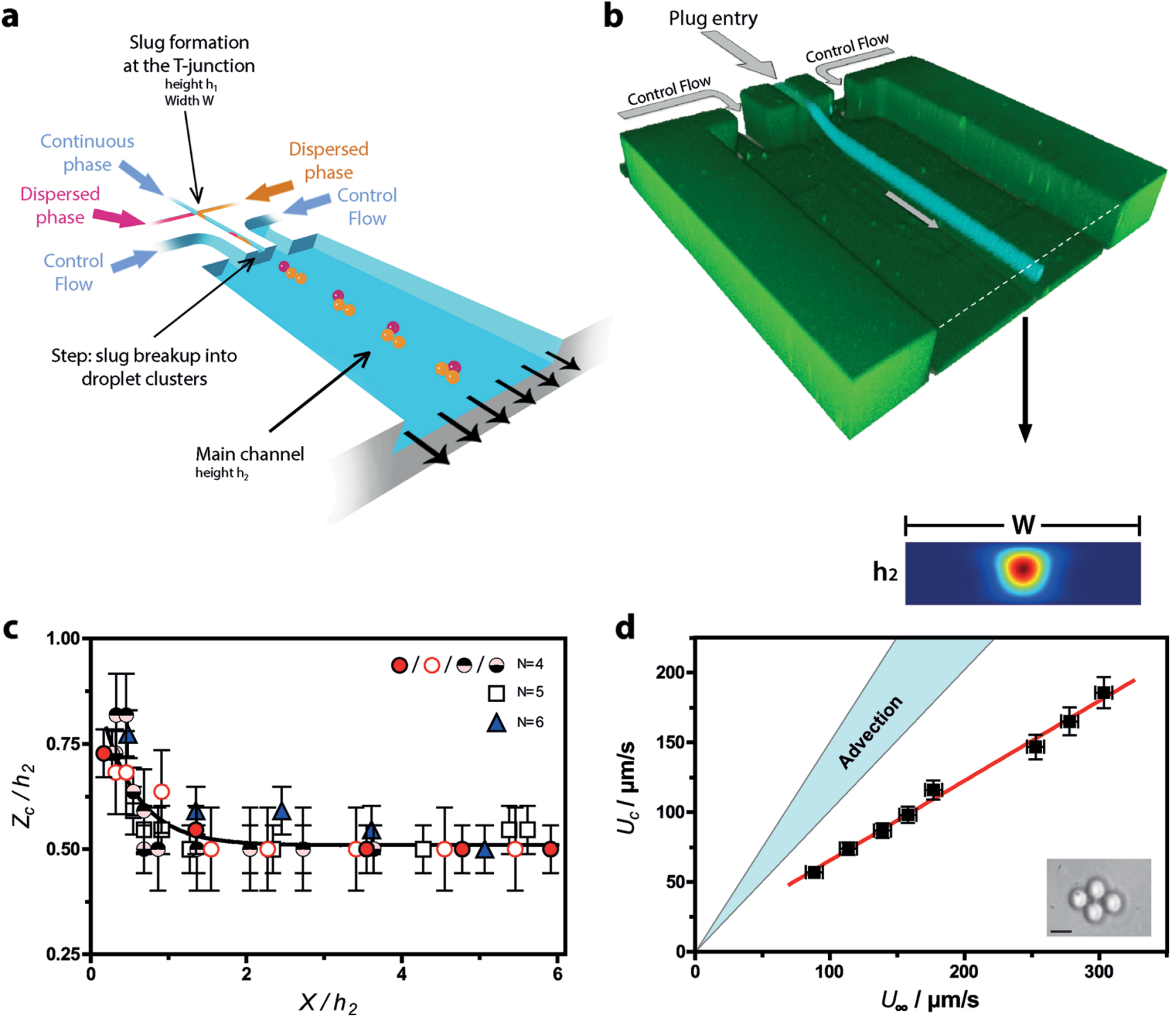
Sketch of the device, flow structure, and droplet positioning in the self‐assembly channel. a) Schematic diagram of the two‐level microfluidic device. b) Confocal image of the system, taken over long exposure times and comparison with COMSOL simulations (Supporting Information), scale bar : 50 μm. c) Cluster position measurements using adaptive focus, for different cluster sizes. The cluster position along the “vertical” *z*‐axis (normal to the bottom wall), normalized by the main channel height *h*_2_, is shown as a function of the streamwise distance *x* to the step normalized in the same manner. “*N*” is the number of droplets inside the cluster. All the experiments are performed in PDMS systems, with *h*_1_ = 1 μm, *h*_2_ = 10 μm, and *d* = 5 μm using fluorinated oil, water, and 2% SDS. Different symbols are used for *N* = 4, to indicate that different pressure conditions at the control flow entry have been used. d) Diamond speed *U*_C_ as a function of the mean flow upstream speed *U*_∞_, obtained by dividing the flow‐rate through the main channel by the cross‐sectionnal area. Full lines represent the passive advection hypothesis, in two limiting cases: *U*_c_ = *U*_∞_ (if the cluster obstructed the main channel) and $U_{c}=\frac{3}{2}U_{\infty }$ (if the cluster was pointwise and located on the symmetry axis of the main channel). The PDMS system dimensions are *h*_1_ = 1 μm, *h*_2_ = 22 μm, *w* = 20 μm, and fluorinated oil in water was used with 2% SDS. Scale bar: 5 μm.

As sketched in **Figure** **1**a, the microfluidic device is fabricated using a two‐level lithography.^15^ The thinner channels (heigth *h*_1_ between 0.5 and 10 μm, width *W* between 2 and 50 μm) of our microfluidic device are located upstream. In this part, the immiscible fluids meet at the T‐junction, generating sequences of plugs, either isolated or grouped by pairs of different chemical compositions (see the Supporting Information). Transported by the flow, the plugs arrive at deeper microchannel (“main channel”, height *h*_2_ between 5 and 163 μm) in which they are transported downstream. At the step, the plugs break up into sticky spherical droplets that aggregate into clusters (see Figure 1a). This is a result of the protocol used (see the Experimental Section) where the surfactant concentrations are far above the cmc, thereby favoring adhesive depletion forces.

Two additional flow entries (Control flows in Figure 1b), located at 200 μm from the vertical symmetry plane of the device dilute them, i.e., increase the distance between two successive clusters (Supporting Information).

Depending on the flow conditions, plugs arriving at the step, break up into droplets of identical or different sizes^17^ (see the Supporting Information). The droplets are initially localized close to the top wall of the main channel (see Figure 1a,b).

The plots and images of Figure 1 are obtained for the case of moderate adhesion,^18^, ^19^ i.e., when no salt is added. Here, the aggregates tend to form planar structures that self‐center in the main channel as they travel downstream (see Figure 1b). We observe that clusters are confined in a centered tube, whose axis is parallel to the channel walls (Figure 1b). The optical measurements of Figure 1c confirm this conclusion, as does the COMSOL study of Figure 1b, detailed in the Supporting Information. Figure 1d shows that cluster speeds are significantly below those that they would adopt if they were passively advected by the flow. This effect^20^, ^21^ is presumably due to the cluster friction against the channel walls. Figure 1d also indicates that in our case, the slowing down effect is significant, in comparison with the case of purely advected isolated droplets.

**Figure 2:**
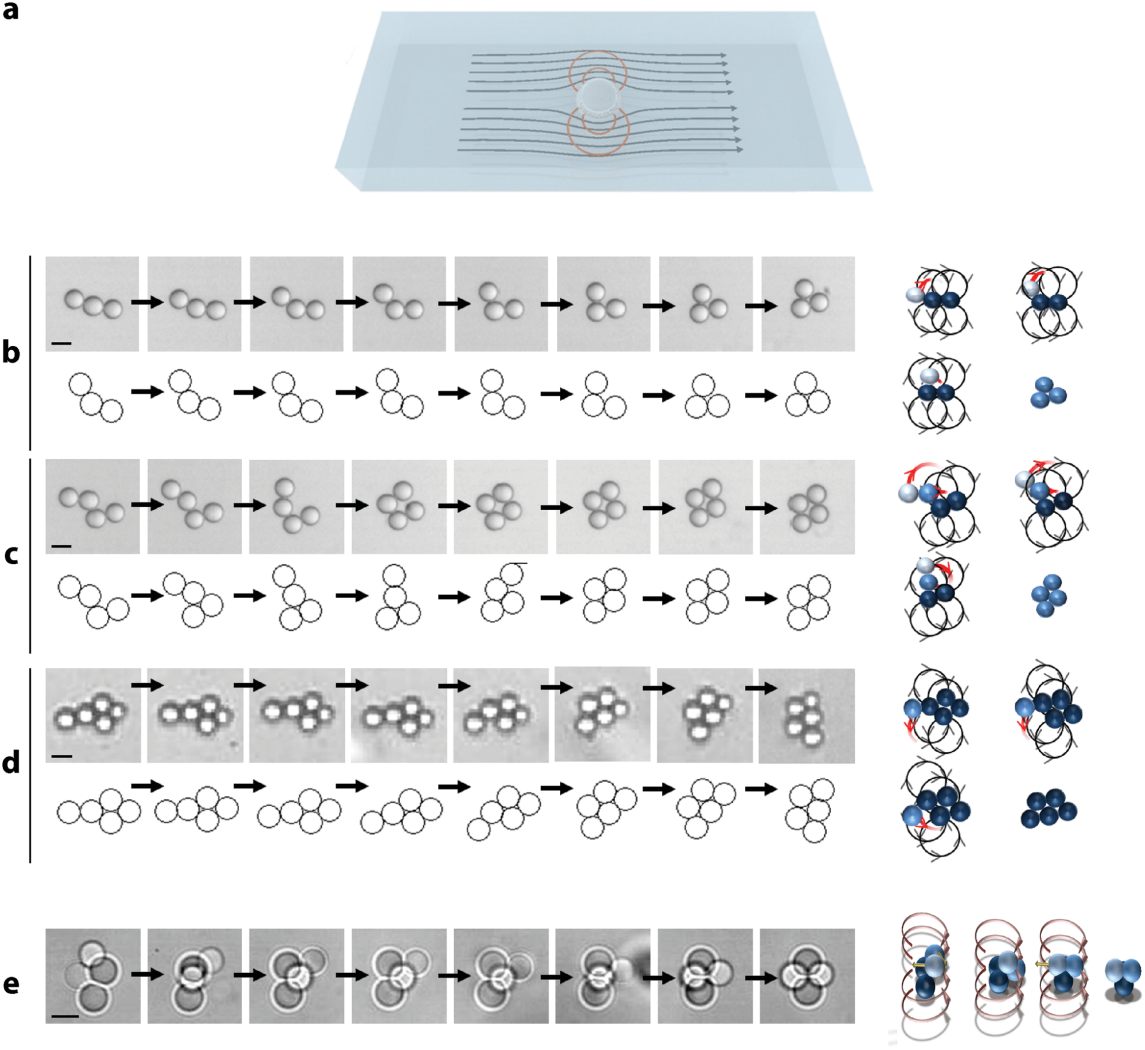
Rearrangement kinetics. a) Schematic view of the flow field associated to an isolated droplet moving in a microchannel. b) Dynamics of a cluster of three droplets evolving toward an equilateral triangular planar structure (see Movie S2 in the Supporting Information). *Top*: Experiment made with fluorinated oil in water, 2% SDS, with droplets 50 μm in diameter. Between each snapshot, the time interval is 0.12 s. Scale bar, 50 μm. *Bottom*: Numerical simulation based on Equation (1), with *Y* = 1.5 – see the definition in the text and time intervals of one characteristic time τ (defined in the text). *Right*: Representation of the recirculations associated to each droplet within the cluster. After 0.84 s, the cluster reaches a steady configuration in which the effect of the recirculations cancel out. c) Same situation for *N* = 4 (*d* = 50 μm, time interval 0.4 s). Scale bar, 50 μm (see Movie S3, Supporting Information) *Y* = 0.8, intervals of 6 τ and d) *N* = 5 (*d* = 5 μm, time interval 1 s). Scale bar, 5 μm. *Y* = 0.8 and intervals of 15 τ. e) *Left*: Kinetics of a tridimensionnal tetrahedron, in the case *N* = 4 (see Movie S5, Supporting Information). The fluids are fluorinated oil in water with 2% SDS and 5% NaCl. Here, *d* = 50 μm, and the time intervals are 0.04 s. Scale bar, 5 μm. *Right*: Representation of the dipolar recirculations leading to the formation of a compact tetrahedron.

*Rearrangement Kinetics*: The droplet clusters reorganize spontaneously as they travel downstream; this phenomenon is displayed in Movies S1, S2, and S3 in the Supporting Information, for clusters including two, three, and four identical droplets. The phenomenon deserves a detailed analysis. In **Figure** **2**b–d, obtained for moderate adhesion, we track 2D clusters moving downstream in the main channel. All the droplet interfaces being well focused, the structures are planar. In Figure 2b,c, the clusters have initially the form of bent chains. After they are formed, they undergo internal rearrangements and eventually adopt symmetric stationary configurations, i.e., a horizontal equilateral triangle for *N* = 3, a diamond for *N* = 4, and a flat isosceles trapezoid for *N* = 5 (Figure 2b–d). The process takes a few seconds to be completed. During the conformational changes, droplets roll alongside each other in the horizontal plane. Eventually the clusters adopt stationary configurations for which, compared to the initial conditions, the number of internal droplet–droplet contacts, *C*, is augmented and the level of symmetry of the structure is increased. Similar comments can be done for the 3D case. Figure 2e, obtained for large adhesion, shows the formation of a compact tetrahedron in a few seconds, within which symmetry has augmented (Movie S5, Supporting Information).

The reorganization process shown in Figure 2b–e is not driven by Brownian fluctuations because it would take days whereas in the experiment. In order to understand this reconfigurational mechanism, we focus on the 2D structures, which, owing to their simplicity, are more amenable to a thorough analysis. As demonstrated in Figure 1b–d, the planar clusters lie in a plane located close to the symmetry axis of the main channel, i.e., in a region where the shear is zero. Although they are advected by an approximately uniform steady flow, they undergo a dynamics that leads to rapid internal rearrangements. We propose here that the physical origin of the phenomenon is linked to the presence of the top and bottom walls of the main channel that slow down the cluster speed. In such circumstances, a dipolar hydrodynamical field develops (see Figure 2a). In fact, in a frame moving with the average speed of the external phase, the clusters move backward, and, owing to mass conservation, the fluid that it must displace to recede recirculates in the forward direction, which gives rise to the dipolar‐like pattern sketched in Figure 2a. This reasoning can be made for each droplet embedded in a cluster. In each aggregate, the horizontal recirculations developed by each droplet exert viscous drags onto their partners, displacing them with respect to each other, therefore provoking configurational changes. In the case of *N* = 3, the droplet located at the rear of the aggregate is subjected to the recirculations generated by its partners, which work at bringing it closer to the center of the doublet they form (see Figure 2b). As the droplet arrives at its final destination, the action of the recirculations cancels out by symmetry, and the configuration becomes stationary. Similar arguments apply for *N* > 3, along with 3D clusters (Figure 2b–e)

## 3 Theoretical Modeling

The mechanism discussed above can be modeled in the 2D case. Similarly as in refs.,^22^, ^23^, ^24^ we model the droplet‐wall interactions by far‐field pairwise dipolar interactions, noting that this approach remains acceptable at a semiquantitative level when droplets touch each other. We thus model the behaviors of our clusters by the following system of 2D dimensionless equations (see the Supporting Information), placing ourselves in a frame of reference moving with *U*_∞_, taking *R* (the droplet radius) as the reference scale, and *τ* =*R/β(*1−*β)U*_∞_ (in which *U*_∞_ is the speed at infinity) as the reference time (1)
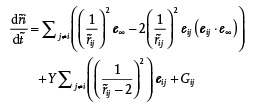
in which $\frac{d\tilde{r}i}{d\tilde{t}}$ is the dimensionless speed of droplet *i* (in which ${\tilde{r}}_{i}$ is its position, and $\tilde{t}$ is the dimensionless time), ***e***_∞_ is the unit vector projected onto the mean flow at infinity, *ß* is the reduction factor of the cluster speed (assumed to be due to friction against the walls, as discussed in the previous sections), ${\tilde{r}}_{ij}$ is the separation distance between droplets *i* and *j*, ***G*** *_ij_* is a repulsive short range term that prevents droplet interpenetration (see the Supporting Information), and *Y* is a dimensionless number given by the following expression

(2) $$Y=\frac{A}{72\pi \eta R^{2}U_{\infty }(1-\beta )}$$

in which *A* is the constant used in the attractive part of the droplet–droplet potential (Supporting Information), and *η* is the external phase viscosity. In Equation (1), the first term of the RHS is the drift caused by the mean flow, the second the dipolar droplet–droplet interaction, the third the adhesive term, and the last one a short range repulsive term that prevents interpenetration. The dimensionless number *Y*, which stands as the unique control parameter of the problem, is new. On physical grounds, it represents the ratio of the adhesive droplet–droplet forces over the dipolar forces. At small *Y*, adhesion is small and droplets separate out; while at large *Y*, droplets stick together permanently.

Solutions to Equation (1), obtained with the initial conditions of Figure 2, are shown in Figure 2b–d for *Y* > 0.1. The agreement between theory and experiment is remarkable. In all cases, the sequences of events calculated with Equation (1) coincide well with the experiment (see Movies S6, S7, and S8 in the Supporting Information). The model demonstrates that a dipolar interaction, coupled to adhesion conditions, nurtures an interesting configurational process, that leads to the formation of symmetric structures. The excellent agreement between the model and the observations indicates that we have captured the physical mechanism at work in the experiments.

**Figure 3:**
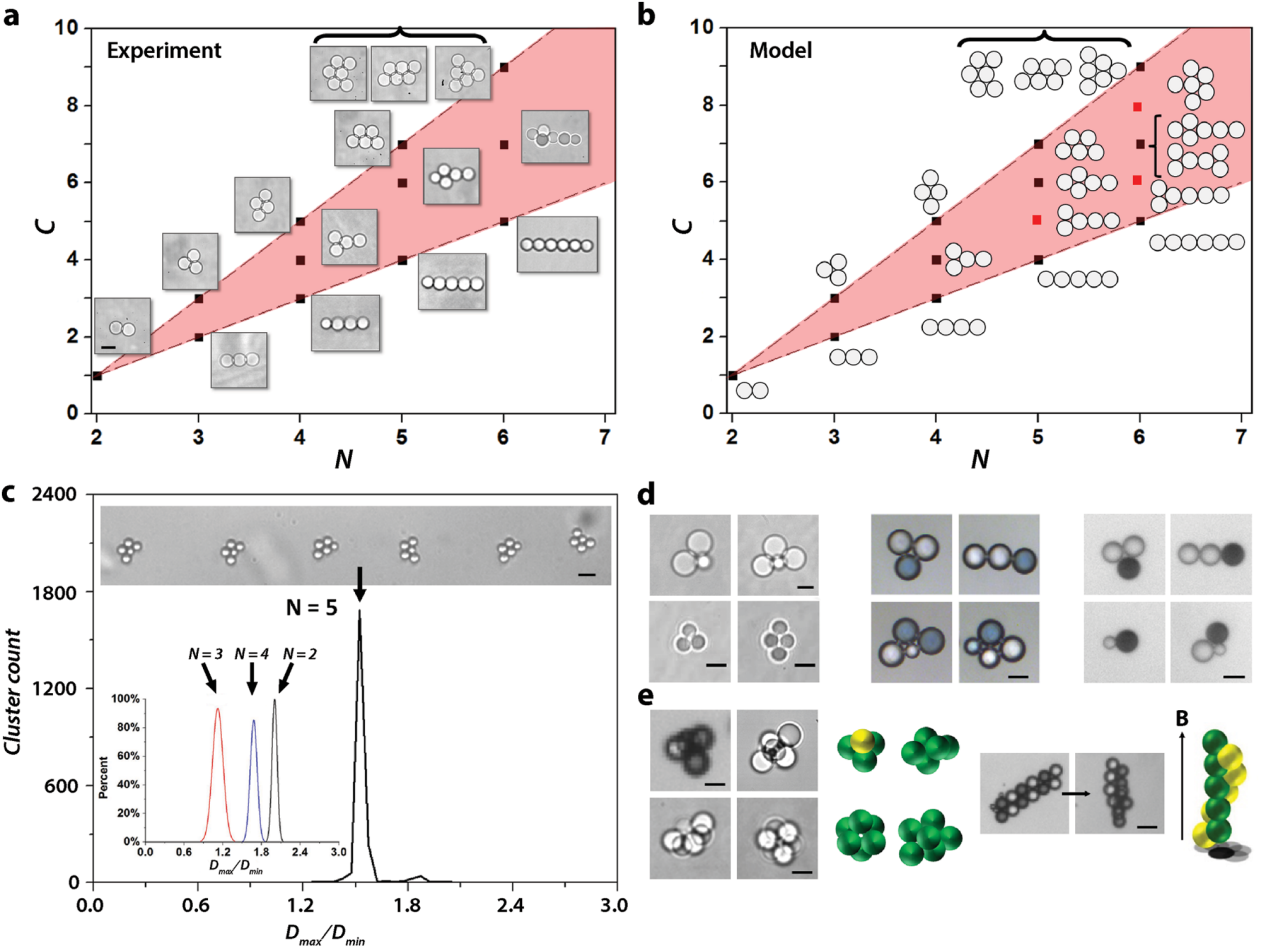
Stationary configurations. a) Observed cluster morphologies. *C* represents the number of droplet–droplet contacts, *N* is the number of droplets per cluster. Scale bar is 5 μm. b) Simulation of the stationary morphologies, based on Equation (1). Red dots represent structures not observed experimentally. c) Histogram of pentamer clusters aspect ratio λ = *D*_max_ */D*_min_ for a population of 2134 clusters, including 5 droplets, 5 μm in diameter, in which *D*_max_ is the Feret maximum diameter and *D*_min_ the minimum one. The coefficient of variation (i.e., the quantity 100 *σ*/<*λ*>) of the major cluster population is ≈2%, where *σ* is the standard deviation and <*λ*> is the statistically averaged aspect ratio. Scale bar: 10 μm*. Inset*: Distributions of the Feret aspect ratios for populations of 152 doublets, 382 triangles, and 217 diamonds, all with droplet diameters of 50 μm. d) A collection of clusters possessing anisotropic structures. *Left array*: Shape anisotropies, (left) AB_2_ type structure, (right) AB_3_ trigonal pyramid; (bottom) micrometer‐size clusters, Scale bars 5 μm. *Central array*: Chemical anisotropies for *N* = 3 (compact AB_2_ and linear AB_2_ type structures) and combinations of chemical and geometrical anisotropies (heterogeneous quadrimers). Scale bar, 50 μm. *Right array*, obtained with fluorinated oil in water, with 2% SDS; various structures with droplets incorporating ferromagnetic particles (Mickey mouse shape AB_2_, linear AB_2_, heterodimer, heterotrimer). Scale bar, 50 μm. e) 3D clusters. *Left array*: Polyhedron clusters with *N* = 4, 5, 6, and AB_3_ tetrahedron structure, with three magnetic droplet (black) capable of developing a directional, localized magnetic bonding. Scale bar, 50 μm for tetrahedron structure and 5 μm for the others clusters (with *N* = 5, 6). *Center left*: Representations of the 3D structures using yellow for the magnetic and green for the nonmagnetic droplets. *Center Right*: Effect of an homogeneous magnetic field on an heterogenous magnetic cluster. Magnetic droplets align with magnetic field. (Scale bar, 40 μm). *Right*: Schematic representation of the final structure.

*Stationary Structures*: The stationary planar structures we observed are displayed in **Figure** **3**a. The structures have different shapes—T, crosses, diamonds, trapezoids, triangles… which all exhibit a mirror symmetry. These structures are classified as function of the number *N* of droplets they include and the number *C* of droplet–droplet contacts they achieve. All the configurations are confined in a pink triangular‐shaped domain, delimited by two lines (*C* = *N*−1 and *C* = 2*N*−3) corresponding, respectively, to chains and compact structures. Inside the pink triangle, apart from three exceptions, we have succeeded, by varying the flow conditions, to fill the space, i.e., achieve all possible contact numbers. As long as *N* is smaller than 6, the structures are unique for a fixed pair (*N*, *C*). However, similarly as in three dimensions, degeneracies are observed for *N* = 6 (see Figure 3a). The experimental diagram of Figure 3 is well reproduced in our model (See Figure 3b).

The notion of monodispersivity is demonstrated in Figure 3c: We obtain a 2% geometrical dispersivity for 2000 diamond and comparable performances for *N* = 2, 3, and 4 (see the histograms in Figure 3c). The notion of high throughput is illustrated by the production of 1.2 × 10^5^ monodisperse trimers in 245 min (see Movie S10, Supporting Information). This number would allow to make a sample of 20 μm × 2 mm × 2 mm, i.e., whose size is sufficient to expect a “macroscopic” behavior.

The mechanism of cluster reconfiguration we described above is not restricted to identical droplets. Figure 3d shows that, with our approach, stationary structures including unlike droplets, with heterogeneous chemistries and shapes, and different magnetic properties, can be produced under control (see Movie S4, Supporting Information). Structures with unlike droplets are obtained by working with plugs that break up into sequences of droplets of different sizes, while structures with different chemical compositions are obtained by driving plug pairs of different compositions into the main channel. Structures with magnetic droplets are obtained with magnetic nanoparticles injected in one of the dispersed phases. Figure 3e shows a magnetic cluster undergoing a morphological change as a magnetic field is applied, giving rises to a spiral. Our technique also produces 3D clusters essentially close packed polyhedrons, homo or heterogeneous (see Figure 3e).

## 4 Solidification

**Figure 4:**
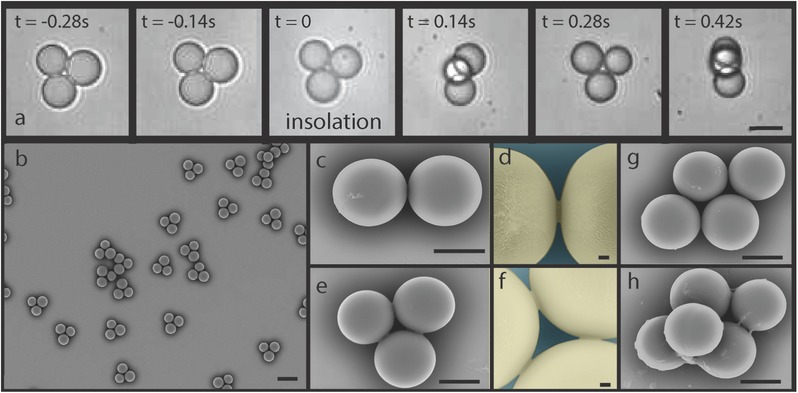
Solidified clusters. a) Series of micrographs taken during solidification of the inner‐phase monomer. The video (Video S9, Supporting Information) shows in‐line insolation and polymerization of clusters in the main channel. After polymerization, clusters spin as they move downstream (channel height: 55 μm). Scale bar, 20μm. b) Electron micrograph of triplet clusters. After polymerization, triplets are collected, washed with water, dried, and observed. Scale bar, 30 μm. c) Electron micrograph of one doublet cluster. Scale bar, 10 μm. d) Close up on the polymerized bridge of previous doublet. False color. Scale bar, 1 μm. e) Electron micrograph of one triplet cluster. Scale bar, 10 μm. f) Close up on the polymerized bridges of previous triplet. Bridges permanently hold solidified droplets together. False color. Scale bar, 1 μm. g) Electron micrograph of one quadruplet (diamond‐shaped) cluster. Scale bar, 10 μm. h) Electron micrograph of a 3D quintuplet cluster. Scale bar, 10 μm.

We successfully solidified these structures in situ by using acrylate based monomers, and adjusting solubilities so that polymerization occurs both in the disperse and the continuous phase. **Figure** **4** shows a situation where the dispersed phase is a solution of diethylene glycol diacrylate with 5% 2‐Hydroxy‐2‐methylpropiophenone and the continuous phase is water with 2% SDS pre‐equilibrated with the previous solution. To solidify the clusters, we illuminate locally the main channel, using a technique similar to reference.^25^ As the clusters penetrate in the illuminated region (see Figure 4a, at *t* = 0 s and Video S9, Supporting Information), they solidify in less than 200 ms (Figure 4a; Video S9, Supporting Information). The solubility of the monomers and the initiators in the continuous phase leads to form polymerized bridges, anchored to the solid particles (Figure 4c,d). These bridges, a few hundred nanometers high, provide strong mechanical cohesion of the entire structure. Figure 4b,c shows scanning electron microscope pictures of solid clusters obtained in this way, along with close up views of the bridges.

## 5 Conclusion

We show here that microfluidics allows to design colloidal structures one by one, under high throughput conditions (10^5^ trimers in four hours per line), with excellent monodispersivity (typically 2%) and a broad range of configurations (compact or not, anisotropoic, magnetic,...). Some of them (T, crosses, tetahedron with magnetic heads), difficult to obtain with the current techniques, could be assembled directly (i.e., without chemical functionalization) to create noncompact materials with potentially interesting optical properties. The magnetic tetrahedrons of Figure 4e could also be directly assembled in the presence of a magnetic field to form noncompact crystals, interesting to evaluate. Indeed, adding local chemical functionalization, in situ or ex situ, may also allow to create interesting materials. Increasing flow rates can be done by parallelization, which takes advantage of microfluidics scale. We can expect a gain of two orders of magnitude with a hundredfold parallelized device. In this configuration, the formation of 10^7^ colloidal “molecules” should be feasible in one day. This is enough to create a 20 μm × 1 mm × 1 mm sample that expresses a “macroscopic” behavior. Also, by reducing the device dimensions, droplet sizes down to 400 nm can be obtained,^16^ which may be interesting for creating photonic materials operating in the visible range. To conclude, the present work opens new routes for designing building blocks enabling the creation of colloidal materials.

## 6 Experimental Section

*Microfluidic Devices*: The microfluidic devices were made by standard soft photolithography and replica‐molding techniques from polydimethylsiloxane (PDMS). The molds were prepared using photolighography of a UV‐curable epoxy (SU8 20XX series, Microchem). They consist of two‐layers structures with different heights. The first layer includes one or several T‐junctions and is followed by a shallow terrace. The depths of the thin channels (*h*_1_) vary between 1 to 10 μm and their widths between 10 to 100 μm while those of the collecting channels, i.e., main channel (*h*_2_) vary between 22 and 163 μm, with a width of 600 μm.

*Fluids and Surfactants*: Different formulations were used: Direct O/W emulsions and inverte W/O emulsions. To produce oil in water structures, fluorinated oil (FC3283, 3M) was used as the dispersed phase and water with surfactant sodium dodecyl sulfate (SDS) ([c] vary between 0.5 and 10 CMC) as the continuous phase. In another case, deionized water was used as the dispersed phase and mineral oil with Span80 (2%) was used as the continuous phase. The formulations with surfactants above the CMC develop adhesive forces between droplets, thanks to depletion forces generated by the presence of micelles. In the meantime droplet coalescence was prevented, owing to the presence of a stable film between the droplets. By adding salt in the O/W emulsion, the repulsive electrostatic barrier was lowered and adhesion between droplets was substantially enhanced. This is reflected by a flattening out of the interfaces at the droplet–droplet contact point.

*Hybrid Clusters Chemical Composition*: Two kinds of formulations were used to produce hybrid clusters. Methylene blue (C_16_H_18_N_3_SCl), a blue ionic dye, was added at high concentration in the dispersed aqueuse phase. It yields a blue solution when dissolved in water at room temperature. The surface tension water/air was lowered from 72 to 60 mN m^−1^ at high concentrations of methylene blue. Mineral oil with 2% surfactant Span 80 was used as carrier fluid in this case. As for the preparation of the magnetic clusters, two different kinds of superparamagnetic particles were used. The first kind consists of aqueous dispersion of microspheres (magnetic core (maghemit Y − Fe_2_O_3_) and silica matrix), with thiol group grafted to their surfaces (purchased from Chemicell GmbH). The size of particles is around 500 nm. The second type of colloidal particles consists of magnetite Fe_3_O_4_ nanoparticles of 40 nm which is dispersed already in octane (80%) (purchased from Ademtech) and which we can further dilute with mineral oil to obtain 0.01 %v/v of ferrofluid in mineral oil. The solution was stabilized by 2% Span80 in the organic phase. Mineral oil with 2% Span 80 was used as carrier fluid in the previous aqueous dispersion and water with 2% SDS for the latter.

*Fluid Driving and Measurement Equipement*: To drive the fluids, pressure sources (MFCS Fluigent) or syringe pumps NEMESYS were used. By using an integrated flowmeter in the case of pressure sources, the flow‐rates of the external phase injected in the different entries could be measured. Throughout the experiments, a range of flow rates varying between 5 and 100 μL min^−1^ was spanned. The droplet motions were recorded with a fast camera (Photron) through an inverted microscope (Zeiss or Leica). Image processing was used to determine the droplets characteristics.

*Adaptive Focus z Position Measurement*: By using the fully automated Leica microscope system with Adaptive Focus Control (AFC), the measurement of the vertical position (*z* coordinate) of the clusters could be performed. These measurements were made on a microfluidic device with *w* = 20 μm, *h*_1_ = 1 μm, and *h*_2_ = 22 μm. After the detection of the floor *(z* = 0), we span the height of the microfluidic main channel (*h*_2_ = 22 μm), up to the ceiling, plot intensity profiles and localize the maximum to determine the cluster “altitude” *z*. The process being reproducible, averaging over many clusters was carried out.

*Microscope Confocal Imaging*: A stationary train of clusters made of aqueous droplets in mineral oil was produced. To improve the quality of visualization, the dispersed phase was mixed with fluorescein isothiocyanate dextran. Rhodamine B red dye (6 × 10^−3^% in aqueous solution), was infused into the channels and washed before the experiments. This dye permeates the PDMS matrix. Fluids were pumped into the devices through PEEK tubing using a pressure controller (MFCS Fluident). The microfluidic system was characterized by *w* = 50 μm, *h*_1_ = 10 μm and *h*_2_ = 163 μm. A series of experiments with different flow conditions was performed, with pressures at the control entries varying from 250 to 700 mbar. Because of a slow exposure time, clusters could not be resolved at the individual level; their averaged trajectories form a florescent tube on Figure 1a.

*Solidification*: Polymer solution was obtained by mixing 5 g diethylene glycol di‐acrylate (Sigma) and 0.25 g 2‐Hydroxy‐2‐methylpropiophenone (Sigma). Water with 2% SDS pre‐equilibrated with the previous solution was used as continuous phase. A 100 W HBO Mercury lamp served as the source of UV. A filter set that provides wide UV excitation (19 000 set, Chroma) was used to select light of desired wavelength. Photomask (César graphique, Paris) was inserted into the field‐stop of the microscope (Zeiss Axio Vert) to create a UV beam inside the chip. By passing through the beam, clusters became solid and droplets were stuck to each other inside the same cluster (Video S9, Supporting Information). PDMS system with *W* = 50 μm, *h*_1_ = 4 μm, *h*_2_ = 55 um was used to produce droplets of 20 μm diameter.

## Supporting information

As a service to our authors and readers, this journal provides supporting information supplied by the authors. Such materials are peer reviewed and may be re‐organized for online delivery, but are not copy‐edited or typeset. Technical support issues arising from supporting information (other than missing files) should be addressed to the authors.

Supplementary

Supplementary

Supplementary

Supplementary

Supplementary

Supplementary

Supplementary

Supplementary

Supplementary

Supplementary

Supplementary

## References

[1] A. Van Blaaderen , Science 2003, 301, 470. 12881559 10.1126/science.1087140

[2] F. Li , D. P. Josephson , A. Stein , Angew. Chem. Int. Ed. 2011, 50, 360. 10.1002/anie.201001451 21038335

[3] O. D. Velev , S. Gupta , Adv. Mater. 2009, 21, 1897.

[4] E. Duguet , A. Désert , A. Perro , S. Ravaine , Chem. Soc. Rev. 2011, 40, 941. 21212874 10.1039/c0cs00048e

[5] S. Sacanna , D. J. Pine , Curr. Opin. Colloid Interface Sci. 2011, 16, 96.

[6] S. C. Glotzer , M. J. Solomon , Nat. Mater. 2007, 6, 557. 17667968 10.1038/nmat1949

[7] G.‐R. Yi , D. J. Pine , S. Sacanna , J. Phys. Condens. Matter 2013, 25, 193101. 23611897 10.1088/0953-8984/25/19/193101

[8] Y. Wang , Y. Wang , D. R. Breed , V. N. Manoharan , L. Feng , A. D. Hollingsworth , M. Weck , D. J. Pine , Nature 2012, 491, 51. 23128225 10.1038/nature11564

[9] Y. K. Koh , C. H. Yip , Y.‐M. Chiang , C. C. Wong , Langmuir 2008, 24, 5245. 18435554 10.1021/la800702d

[10] S. Wong , V. Kitaev , G. A. Ozin , J. Am. Chem. Soc. 2003, 125, 15589. 14664606 10.1021/ja0379969

[11] N. B. Crane , O. Onen , J. Carballo , Q. Ni , R. Guldiken , Microfluid. Nanofluid. 2013, 14, 383.

[12] G.‐R. Yi , T. Thorsen , V. N. Manoharan , M.‐J. Hwang , S.‐J. Jeon , D. J. Pine , S. R. Quake , S.‐M. Yang , Adv. Mater. 2003, 15, 1300.

[13] E. Gerstner , Nat. Phys. 2011, 7, 98.

[14] T. M. Schneider , S. Mandre , M. P. Brenner , Phys. Rev. Lett. 2011, 106, 094503. 21405629 10.1103/PhysRevLett.106.094503

[15] F. Malloggi , N. Pannacci , R. Attia , F. Monti , H. Willaime , B. Cabane , F. Poncet , Langmuir 2010, 26, 2369. 19916489 10.1021/la9028047

[16] H. Willaime , V. Barbier , L. Kloul , S. Maine , P. Tabeling , Phys. Rev. Lett. 2006, 96, 054501. 16486936 10.1103/PhysRevLett.96.054501

[17] P. Poulin , F. Nallet , B. Cabane , J. Bibette , Phys. Rev. Lett. 1996, 77, 3248. 10062171 10.1103/PhysRevLett.77.3248

[18] P. Poulin , J. Bibette , Phys. Rev. Lett. 1997, 79, 3290.

[19] E. Evans , E. Sackmann , J. Fluid Mech. 1988, 194, 553.

[20] A. Eri , K. Okumura , Soft Matter 2011, 7, 5648.

[21] T. Beatus , T. Tlusty , R. Bar‐Ziv , Nat. Phys. 2006, 2, 743.

[22] W. E. Uspal , P. S. Doyle , Phys. Rev. E 2012, 85, 016325. 10.1103/PhysRevE.85.016325 22400675

[23] B. Shen , M. Leman , M. Reyssat , P. Tabeling , Exp. Fluids 2014, 55, 1.

[24] D. Dendukuri , D. C. Pregibon , J. Collins , T. A. Hatton , P. S. Doyle , Nat. Mater. 2006, 5, 365. 16604080 10.1038/nmat1617

[25] L. Shui , A. Van Den Berg , J. T. Eijkel , Microfluid. Nanofluid. 2011, 11, 87.

