# Supplementary material for: Designing Colloidal Molecules with Microfluidics
Source: Adv Sci (Weinh). 2016 Apr 25;3(6):1600012. doi: 10.1002/advs.201600012 (PMC5080599; doi:10.1002/advs.201600012)
Supplement: Supplementary file 1 — Supplementary [file ADVS-3-0a-s001.pdf]

Copyright WILEY-VCH Verlag GmbH & Co. KGaA, 69469 Weinheim, Germany, 2016.

ADVANCED SCIENCE
Open Access

Supporting Information

for *Adv. Sci.,* DOI: 10.1002/advs.201600012

Designing Colloidal Molecules with Microfluidics

*Bingqing Shen, Joshua Ricouvier, Florent Malloggi, and Patrick Tabeling**

Supporting Information

Supplementary Material 1 : Droplet number per cluster

*Bingqing Shen, Joshua Ricouvier, Florent Malloggi and Patrick Tabeling**

When the plugs produced at the T-junction reach the step, they break up into droplets. The number of droplet per cluster that results from this process depends on the flow-rates of the two phases. Here 1). There exist plateaus on which the droplets are identical and, thereby, *N* is an integer. In between, the clusters include several droplets of different sizes the droplet number *N* is defined as the total cross-sectional area of the cluster divided by the area of the larger droplet. For a fixed flow-rate of the external phase, this number increases with the plug volume, in a stepwise manner (see Supplementary Figure – several identical, one smaller -, giving rise to snowman shapes, or heterogeneous chains. For such cases, *N* is a non integer number. In fact, this evolution has the form of a devil's staircase, reminiscent of the nonlinear interaction between two frequencies, one being associated to the plug generation at the T junction, and the other to the droplet formation at the step.[25]

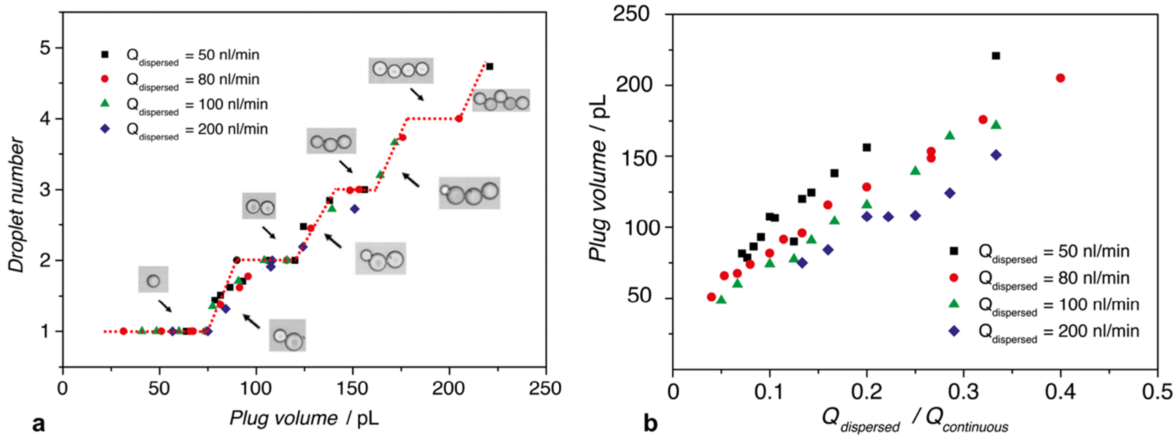

Figure S1: Devil's staircase of droplets production. Droplet number (defined as the total cross-sectional area of the cluster divided by the area of the larger droplet) as a function of plug volume, for a fixed flow-rate of the external phase (w=50µm, h_1=10µm). (b) Plug volume as a function of $Q_{disperse}/Q_{continuous}$ for microfluidic systems (W=50µm, h_1=10µm).

Supplementary Material 2: Role of the control flow.

*Bingqing Shen, Joshua Ricouvier, Florent Malloggi and Patrick Tabeling**

The control flow influences the initial configuration of the cluster at the step, along with the distances between successive clusters in the SA channel. This is shown in the Figure below, for triplets. Here we maintain the pressures of the dispersed and continuous phases constant (resp. equal to 38 and 11 mbar), and we increase the pressure P_C at the control entries.

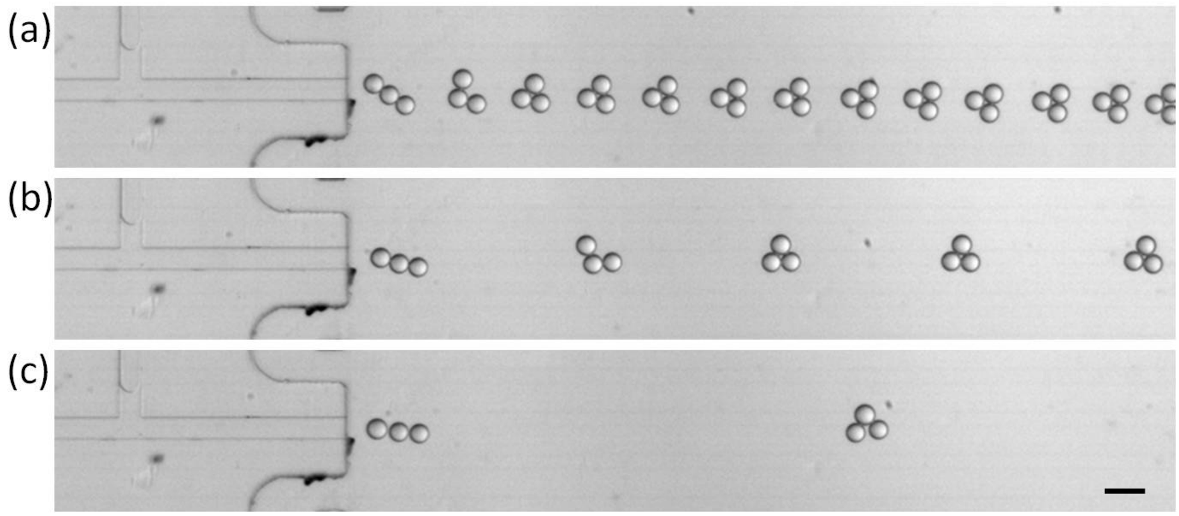

Figure S2: Spacing control between the clusters in SA channel**.** Different configurations observed at various P_C, in a system where W=50μm, h_1=10μm, h_2=160μm. (a) PC= 20mbar. The distance between clusters is 100μm. (b) PC= 80mbar. The distance between clusters is 400μm. (c) PC= 120mbar. The distance between clusters is 1100 μm. The scale bar is 100μm. As the pressure at the control entries raises up, the orientation of the cluster at the entry tends to align with the mean flow, and, in the meantime, the distances between two successive trimers in the SA channel increase, as the result of mass conservation.

Supplementary Material 3: Numerical simulation.

*Bingqing Shen, Joshua Ricouvier, Florent Malloggi and Patrick Tabeling**

We used the commercial COMSOL Multiphasics software to simulate the flow in the microfluidic device shown in Figure 1a-1b. From the velocity field we determine the streamline pattern. The streamlines plotted in Supplementary Figure 3-1 originate from the central inlet channel. The streamlines associated to the largest speeds, and therefore carrying most of the flux, tend to self-center beyond a transient region whose size is on the order of the SA channel height h_{SA}. These patterns agree well with the experiment (see Figure 1b).

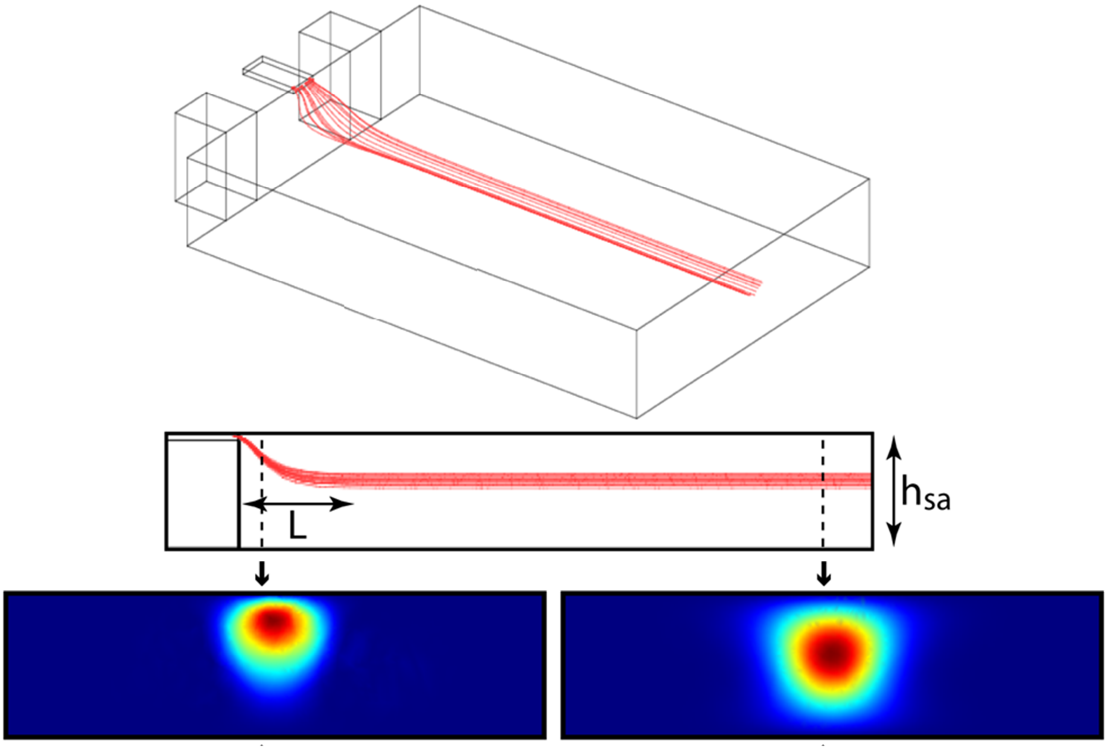

Figure S3: Streamlines originating from the central inlet channel.

Supplementary Material 4: Theory

*Bingqing Shen, Joshua Ricouvier, Florent Malloggi and Patrick Tabeling**

The model we consider is based on the system of equations obtained in Reference[22](Shen, B., Leman, M., Reyssat, M. & Tabeling, P. Dynamics of a small number of droplets in microfluidic Hele–Shaw cells. *Exp. Fluids* **55,** 1–10 (2014).). In this paper, the two dimensional dynamics of a system of *N* identical droplets, with speeds smaller than the upstream flow, was modelled. In our case, we must modify these equations, by adding two terms, one representing the adhesion forces between identical droplets (see for instance *Foundations of Colloidal Science*, Snd Ed., R.Hunter, p 542), and the other corresponding to the short-range repulsive forces that prevents droplet interpenetration. With such modifications, the system reads:

$$\boldsymbol{U}_i = \beta \boldsymbol{U}_\infty + \beta \sum_{j \neq i} (1-\beta) \left(\left(\frac{R}{r_{ij}}\right)^2 \boldsymbol{U}_\infty - 2 \left(\frac{R}{r_{ij}}\right)^2 \boldsymbol{e}_{ij} (\boldsymbol{e}_{ij}.\boldsymbol{U}_\infty) \right) + \sum_{j \neq i} \frac{A\beta}{72\pi\eta} \left(\left(\frac{1}{r_{ij} - 2R}\right)^2 + F_{ij} \right) \boldsymbol{e}_{ij}$$

in which $\boldsymbol{U}_i$ is the speed of droplet *i* (with $\boldsymbol{r_i}$ the center's position, *R* its radius), $\boldsymbol{e}_\infty$ the unit vector projected onto the mean flow at infinity, β the reduction factor of the cluster speed (due to friction against the wall, as discussed in the paper), r_{ij} the separation distance between droplets *i* and *j*, *A* a constant, η the external phase viscosity, F_{ij} a short range repulsive term that prevents droplet interpenetration.

To obtain the dimensionless equations (1), one introduces the following dimensionless quantities.

$$\widetilde{\boldsymbol{U}_i} = \frac{U_i}{\beta(1-\beta)U_\infty},\ \widetilde{\boldsymbol{r}_{ij}} = \frac{r_{ij}}{R}, \widetilde{\boldsymbol{r}_i} = \frac{r_i}{R} \text{ and } \tilde{t} = \frac{\beta(1-\beta)U_\infty t}{R}$$

in which *t* is the physical time. Placing ourselves in the frame of reference $\beta\boldsymbol{U}_\infty$, and adimensionalizing the above equations, one gets the following system:

$$\boldsymbol{U}_i = \beta \boldsymbol{U}_\infty + \beta \sum_{j \neq i} (1-\beta) \left(\left(\frac{R}{r_{ij}}\right)^2 \boldsymbol{U}_\infty - 2 \left(\frac{R}{r_{ij}}\right)^2 \boldsymbol{e}_{ij} (\boldsymbol{e}_{ij}.\boldsymbol{U}_\infty) \right) + \sum_{j \neq i} \frac{A\beta}{72\pi\eta} \left(\left(\frac{1}{r_{ij} - 2R}\right)^2 + F_{ij} \right) \boldsymbol{e}_{ij}$$

with $\boldsymbol{G}_{ij}$ a dimensionless vector characterizing short range repulsive forces for droplet *i*, at time $\tilde{t}$. This vector bears on F_{ij}. The dimensionless number that naturally emerges from these equations is

$$Y = \frac{A}{72\pi\eta R^2\, U_\infty (1-\beta)}$$

This number represents the importance of the adhesion forces that maintain the droplets within the cluster, in comparison with the dipolar forces that reorganize them. The two forces

never cancel out, but work in a complementary manner: the former is oriented along the droplet separation vectors while the latter develops component normal to it.

VIDEO LIST

*Bingqing Shen, Joshua Ricouvier, Florent Malloggi and Patrick Tabeling**

Movies S1 - 3: Kinetics of clusters formation
Fluorinated oil droplets are suspended in an aqueous solution containing surfactant (2% w/w SDS). Movies are acquired at 25 fr/s and played at a rate slowed down 2.5 times. Scale bar, 100 µm.

Movie S4: Kinetics of hybrid clusters formation
Alternating transparent and blue plugs are formed at the T-junctions, and transported at the step. The transparent plug breaks into two spherical droplets while the blue one forms a single droplet. In this way, a heterotrimer is formed. The video is acquired at 50 fr/s and played real time. Scale bar, 100 µm.

Movie S5: 3D tetrahedron self assembly
Fluids are fluorinated oil in water with 2% SDS and 5%NaCl. Here, d=(0µm. Video is recorded at 25 fr/s and played at a rate slowed down 2.5 times

Movies S6-8: Simulation 2D cluster formation.
Numerical simulations were solved using Matlab. Parameters are Y, initial positions of spheres and time lapse between two frames.

- Movie S6
  - Y = 1.5
  - Sphere 1 (10,0.5)
  - Sphere 2 (12,0)
  - Sphere 3 (14,0)
  - $\Delta t = 0.5$
- Movie S7
  - Y=0.8
  - Sphere 1 (10,2.8)
  - Sphere 2 (12,2)
  - Sphere 3 (13,0)
  - Sphere 4 (15,1)
  - $\Delta t = 0.5$
- Movie S8
  - Y=0.8
  - Sphere 1 (10.-0.0001)
  - Sphere 2 (12,0)
  - Sphere 3 (15,0)
  - Sphere 4 (15,-1)
  - Sphere 5 (13.5,1)
  - $\Delta t = 0.5$

Movie 9: Droplet solidification
In line insolation which induce clusters polymerization

Poly-ethylene glycol di-acrylate droplets are suspended in an aqueous solution containing surfactant (2% SDS). This video was acquired at 50 frames/sec and played real time.

Movie 10: High Throughput cluster formation.
We are able to sustain the production of 7 droplets/sec for four hours, assembling 1.2 10^5 cluster. This movie shows an exemple of this throughput during 1 min.
This video was acquired at 16 frames/sec and played at a rate slowed done twice.
